# The Cassiopeia Glomerulus: Stellar Evolution and Renal Homeostasis

**DOI:** 10.1007/s11606-026-10623-z

**Published:** 2026-07-21

**Authors:** Piotr Bramora

**Affiliations:** Department of Physiology, Neurophysiology With the Laboratory of Psychiatry, W. Bieganski Collegium Medicum, Jan Dlugosz University in Czestochowa, Czestochowa, Poland

**Keywords:** Medical humanities, Narrative medicine, Nephrology, Visual metaphor, Glomerulus

At first glance, this sketch presents a chaotic interplay of dense, tangled lines surrounded by a softer, swirling nebula of stardust. However, closer inspection reveals the unmistakable anatomical borders of a renal glomerulus, with the vascular pole clearly visible on the left, opening into a complex capillary network within the suggested bounds of Bowman’s capsule. The dense, black capillary tufts in the center mirror the turbulent gas clouds of the Cassiopeia A supernova remnant. This visual metaphor bridges the micro- and macro-cosmos: just as exploding stars forcefully expel heavy elements essential for the universe’s cosmic balance,^1^ the delicate and dynamic glomerular capillaries tirelessly filter plasma to maintain human homeostasis. Through this abstract lens, the daily, silent work of the kidneys is reframed as an event of almost galactic magnitude, inviting reflection on the profound structural beauty of human biology (Fig. 1).

**Figure 1:**
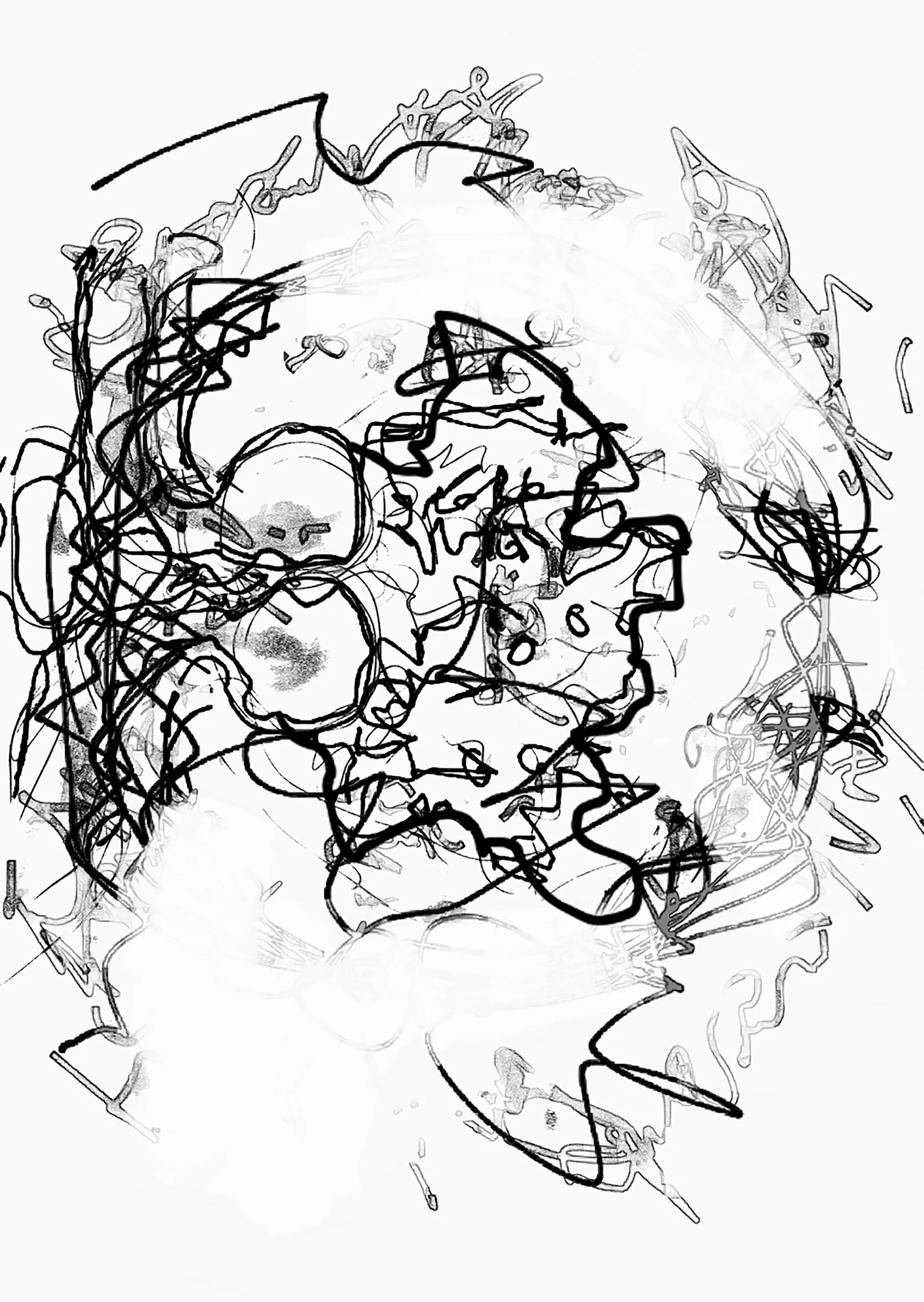
The Cassiopeia glomerulus: stellar evolution and renal homeostasis.

## Data Availability

Data sharing is not applicable to this article as no datasets were generated or analyzed during the current study.

## References

[1] **Holland-Ashford T, Lopez LA, Auchettl K, Temim T, Ramirez-Ruiz E.** Asymmetries of Heavy Elements in the Young Supernova Remnant Cassiopeia A. Astrophys J. 2020;889(2):144. 10.3847/1538-4357/ab64e4.

